# On-line plasma lactate concentration monitoring in critically ill patients

**DOI:** 10.1186/s13054-017-1738-6

**Published:** 2017-06-16

**Authors:** Christian De Tymowski, Sabri Soussi, François Depret, Matthieu Legrand

**Affiliations:** 1 AP-HP, GH St-Louis-Lariboisière, Department of Anesthesiology and Critical Care and Burn Unit, St-Louis Hospital, Assistance Publique- Hopitaux de Paris, Paris, France; 2 University Paris Diderot, Paris, France; 3 UMR INSERM 942, Institut National de la Santé et de la Recherche Médicale (INSERM), Lariboisière hospital Univ Paris Diderot, F-75475 Paris, France

**Keywords:** Lactate, Monitoring, Clearance, Microdialysis

Blood lactate concentration (BLC) is widely used to guide resuscitation in critically ill patients [1]. Muscular microdialysis has previously been used illustrate the importance of the Na/K pump in hyperlactatemia in septic shock patients [2], with BLC reflecting metabolic changes rather than oxygen debt [3]. Microdialysis has now also been applied to intravascular continuous monitoring [4] (Eirus TLC®, Maquet Critical Care, Solna, Sweden). Nevertheless, the potential clinical implications of on-line lactate monitoring are unclear. In this regard, we illustrate the high sensitivity of the device to detect BLC variations and the potential clinical implications of this.

An adult patient presented with a total body surface area burn of 70% after a suicide attempt by self-immolation. A triple-lumen central venous catheter (CVC) with an integrated microdialysis function was placed in the left internal jugular vein. A second CVC was placed in the right femoral vein. Figure 1 shows BLC variation during the first hours after resuscitation. On top of the general trend of BLC assessment, we propose an alternative additional use of continuous BLC monitoring, namely assessment of blood lactate clearance [5]. A short-lasting rise in BLC can be identified in the global trends (circles in Fig. 1a) corresponding to fluid challenges using Ringer lactate (RL; 500 ml, lactate 29 mmol/L, amount of lactate 14.5 mmol). Lactate clearance can be assessed using the flowing formula: Lc (L/kg/h) = D (mmol/kg)⁄AUC (mmol/L/h), where Lc is lactate clearance, D is the amount of L-lactate infused, and AUC is the area under the curve from RL infusion until BLC reaches its initial level. Two RL bolus infusions via the femoral CVC performed with a 2-h interval (Fig. 1b) allowed us to evaluate lactate clearance at 934 and 1172 ml/min, respectively [5]). The AUC was calculated by the trapezoid method, i.e., the sum of BLC means every minute, with the BLC at initiation of the RL infusion used as the reference (Fig. 1c). Lactate production was calculated to be 3518 and 3176 μmol/kg/h, respectively, as lactate production (μmol/kg/h) = blood lactate (mmol/L) × lactate clearance (mL/kg/h) [5]. Inter-measurement variability was 16 and 7%, respectively, for lactate production and clearance.

**Fig. 1:**
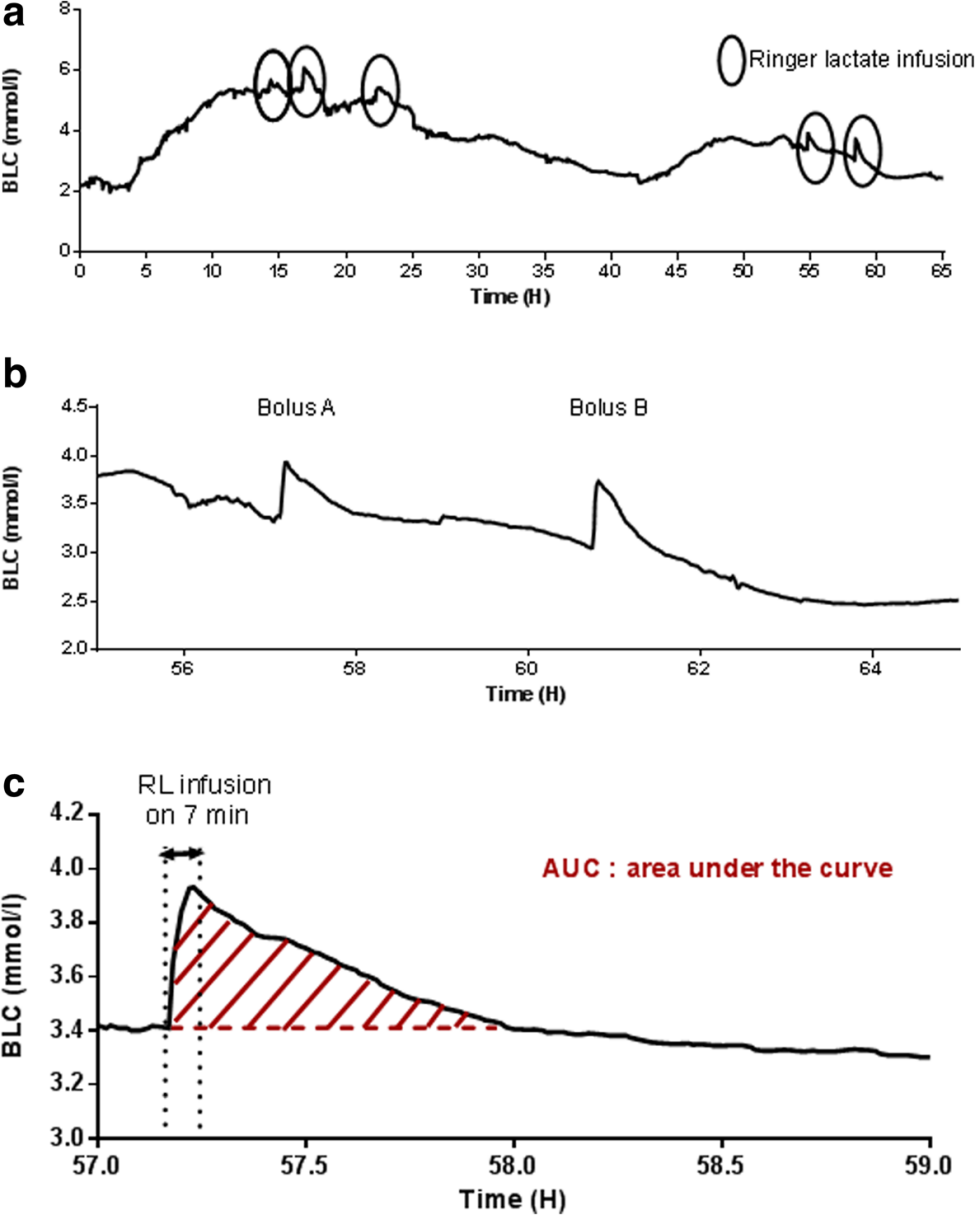
Panel (**a**) Continous plasma lactate monitoring showing changes in lactate level over the first 65 h. Circles indicate Ringer lactate boluses. Panel (**b**) Zoom on changes induced by 2 boluses of Ringer lactate (500ml). Panel (**c**) Area under the curve of plasma lactate during and after infusion of 500 Ringer lactate (Bolus A of panel **b**)

To summarize, this case illustrates that continuous monitoring could allow sensitive detection of rapid and short-lasting changes in BLC after a 500-ml RL infusion. This could be used to assess lactate clearance. Further studies are needed to validate this concept.

## Abbreviations

AUC: Area under the curve
BLC: Blood lactate concentration
CVC: Central venous catheter
D: Amount of L-lactate infused
Lc: Lactate clearance
RL: Ringer lactate
